# Surgical revision for transtibial stump problems following war traumatic amputations: a 2016–2020 cohort from Aleppo, Syria

**DOI:** 10.1186/s12893-026-03603-x

**Published:** 2026-02-16

**Authors:** Qusai Razzouk, Shadi Chachati, Hani Alloush

**Affiliations:** Faculty of Medicine, University of Aleppo, Aleppo, Syria

**Keywords:** Transtibial amputation, Stump revision, Neuroma, Bone spur, Landmine injury, Prosthetic rehabilitation, War trauma

## Abstract

**Background:**

Armed conflicts often lead to a surge in traumatic amputations performed under emergency conditions with limited surgical standards. In Syria, over a decade of war has resulted in a high burden of residual limb complications among amputees, posing major barriers to prosthetic rehabilitation. Despite their clinical significance, long-term surgical outcomes of stump revision in such contexts remain scarcely documented.

**Methods:**

Between 2016 and 2020, a total of 1,450 lower-limb amputees presented to the Syrian Arab Red Crescent Orthopedic clinic in Aleppo. Of these, 110 patients (120 transtibial stumps) who were unable to achieve prosthetic fitting due to residual limb complications underwent surgical revision. Complications included symptomatic neuromas, bony overgrowth, chronic ulcers, scar contractures, and redundant soft tissue. Residual limb length was objectively categorized into five groups (very short, short, optimal, long, Syme), and associations between stump characteristics, trauma mechanism, and complication type were analyzed. All patients were followed prospectively using a standardized multidisciplinary protocol to assess rehabilitation progress.

**Results:**

Bone spurs (45.8%) and symptomatic neuromas (22.5%) were the leading indications for revision surgery. Neuroma incidence was significantly higher in very short and short stumps (*p* < 0.01), while long and Syme-level stumps showed increased rates of bony overgrowth. Landmine and blast injuries were strongly associated with chronic ulcers (60%) and scar contractures (35%). Tailored surgical approaches, including nerve repositioning, bone contouring, and soft-tissue balancing, enabled successful prosthetic fitting in all cases. Functional reintegration was achieved within an average of 95–140 days post-revision.

**Conclusion:**

This prospective cohort highlights the predictable nature of stump complications based on residual limb length and injury mechanism in a war-injured population. Surgical revision, even with basic techniques, proved effective in restoring prosthetic use and function. These findings emphasize the critical importance of structured amputation protocols, optimal stump length preservation, and meticulous nerve handling to reduce long-term complications and achieve sustainable prosthetic rehabilitation in conflict zones and under-resourced surgical environments.

## Introduction

Traumatic limb amputations are a major consequence of armed conflict, with outcomes heavily influenced by the mechanism of injury, surgical context, and subsequent rehabilitation efforts [1]. The global prevalence of limb loss is substantial and projected to rise in coming decades, highlighting the lasting burden of both traumatic and non-traumatic amputations [2]. Recent imaging and clinical studies have revealed a high prevalence of neuromas among transtibial amputees, emphasizing the long-term neurological sequelae of amputation [3–6].

Residual limb complications, including osseous overgrowth and bony spurs, are common among young and active amputees, often impairing prosthetic use and mobility [7, 8]. The coexistence of painful neuromas and skeletal abnormalities further complicates rehabilitation and functional outcomes [9]. These issues are particularly pronounced in conflict zones, where amputations are frequently performed under austere conditions with limited surgical resources [10].

In Syria, prolonged warfare has led to thousands of traumatic amputations, many performed as emergency life-saving procedures in field hospitals or inadequately equipped surgical units. Such conditions often preclude optimal stump formation and nerve handling, predisposing patients to chronic complications [11]. Factors such as stump length, tissue viability, and the energy cost of ambulation further determine the success of rehabilitation and prosthetic adaptation [12, 13].

Advances in reconstructive and nerve-interface techniques have demonstrated promising results in reducing neuroma incidence and post-amputation pain, offering hope for improved long-term outcomes [14–16]. Nonetheless, the demographic most affected—young, otherwise healthy individuals subjected to high-energy war trauma—faces unique lifelong challenges in recovery and reintegration [17, 18].

The present study analyzes a cohort of transtibial amputees managed at the Syrian Arab Red Crescent Orthopaedic Clinic in Aleppo. Our objectives were to describe the demographic characteristics, classification of residual limb length, patterns of stump complications, surgical revision techniques, and rehabilitation outcomes. Documenting these findings is essential not only to preserve clinical lessons learned under extreme humanitarian conditions, but also to inform pragmatic and effective surgical strategies in both conflict-affected and low-resource medical environments.

## Methods

### Study design and cohort

This study is a prospective cohort analysis conducted at the Orthopaedic Clinic of the Syrian Arab Red Crescent in Aleppo, Syria, between June 2016 and November 2020. Patients with war-related transtibial amputations who presented with symptomatic stump complications requiring surgical revision were prospectively included at the time of revision surgery. Data were obtained from standardized medical records routinely collected during humanitarian care.

Within this cohort, 110 patients underwent surgical revision for a total of 120 transtibial amputation stumps, constituting the analytical sample.

Inclusion criteria comprised war-related transtibial amputees presenting to the clinic with clinically significant stump pathology deemed unsuitable for prosthetic fitting and requiring revision surgery. These pathologies included painful neuroma, bony prominence, chronic ulceration, excessive soft tissue, or problematic scarring.

Patients were followed prospectively as part of routine post-revision care using a standardized follow-up protocol conducted by a multidisciplinary team consisting of an orthopaedic surgeon, a physiotherapist, and a prosthetic technician. Follow-up visits were scheduled immediately after surgery, at 1 week, 2 weeks, 1 month, and subsequently at regular intervals over several months to monitor wound healing, resolution of symptoms, prosthetic tolerance, and gait rehabilitation.

### Data collection and variables

Demographic, clinical, and surgical data were systematically recorded. Collected variables included sex, age at the time of revision surgery, limb laterality, mechanism of injury, residual limb length, indication for revision surgery, and temporal variables related to amputation, revision, and rehabilitation.

Residual limb length was measured in centimeters from the tibial plateau to the distal end of the stump and classified into four categories: very short (< 5 cm), short (5–15 cm), optimal (15–25 cm), and long (> 25 cm). Syme amputation was defined as ankle disarticulation with preservation of the heel pad.

Revision indications were categorized based on the pathology that necessitates the intervention and those categories were : excessive soft tissue, problematic scarring, chronic ulceration, symptomatic neuroma and bony prominence.

Diagnostic criteria for stump complications were primarily clinical. Symptomatic neuroma was diagnosed based on localized stump pain associated with paresthesia (numbness or tingling) and/or the presence of a palpable tender nerve mass on physical examination. Bony prominence (spur) was identified by focal distal bone overgrowth causing pressure-related pain and prosthetic intolerance, with plain radiographs used for confirmation when indicated. Ulceration and problematic scarring were diagnosed through direct clinical inspection.

Temporal variables included the interval from initial amputation to revision surgery, the interval from initial amputation to the first attempt at prosthetic fitting, and the time from revision surgery to successful prosthetic fitting.

### Surgical techniques

All revision procedures were performed under general or spinal anesthesia with pneumatic tourniquet control and standard sterile technique. Electrocautery was used for hemostasis, and corrugated drains were placed when clinically indicated.

Surgical techniques were tailored according to the underlying pathology:

- Excessive soft tissue: Redundant muscle and subcutaneous tissue were excised while preserving adequate myocutaneous padding to ensure sufficient coverage and compatibility with prosthetic fitting.
- Symptomatic neuroma: Affected nerves were identified and dissected. The neuroma-bearing nerve stump was ligated proximally and buried into adjacent healthy muscle away from the distal bone end. In total, common peroneal nerve neuromas were treated in 115 limbs and tibial nerve neuromas in 5 limbs.
- Ulceration and scarring: Nonviable tissue and pathological scar were excised completely. Primary wound closure was attempted when local tissue allowed tension-free approximation. In cases with insufficient soft tissue, concomitant shortening osteotomies of the tibia and/or fibula were performed to facilitate closure.
- Muscle Fixation and Management of Bony Prominence: Muscle fixation was routinely performed using a myodesis technique. After radiographic localization of the bony prominence, careful soft-tissue dissection was carried out to adequately expose the involved area. Pathological bony protrusions were excised using osteotomy, and the residual bone was meticulously contoured with a mechanical burr to obtain a smooth, load-tolerant stump end. A sufficient segment of bone was removed to allow tension-free muscular coverage. The muscle was then securely fixed to provide adequate soft-tissue padding over the distal tibia, aiming to reduce the risk of recurrent prominence, skin breakdown, and bone exposure.

**Fig. 1:**
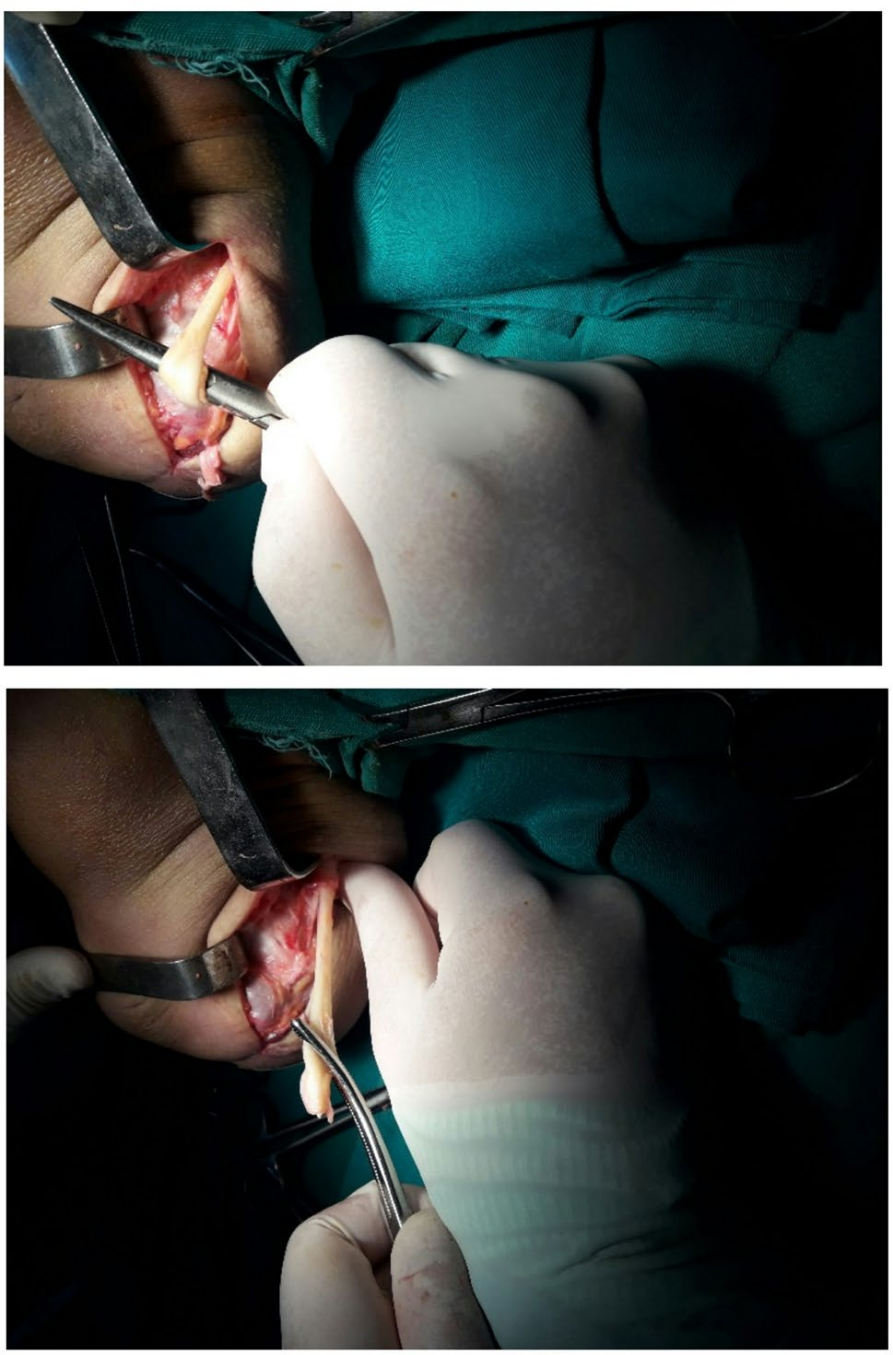
Peroneal nerve adhesion to the amputation scar

**Fig. 2:**
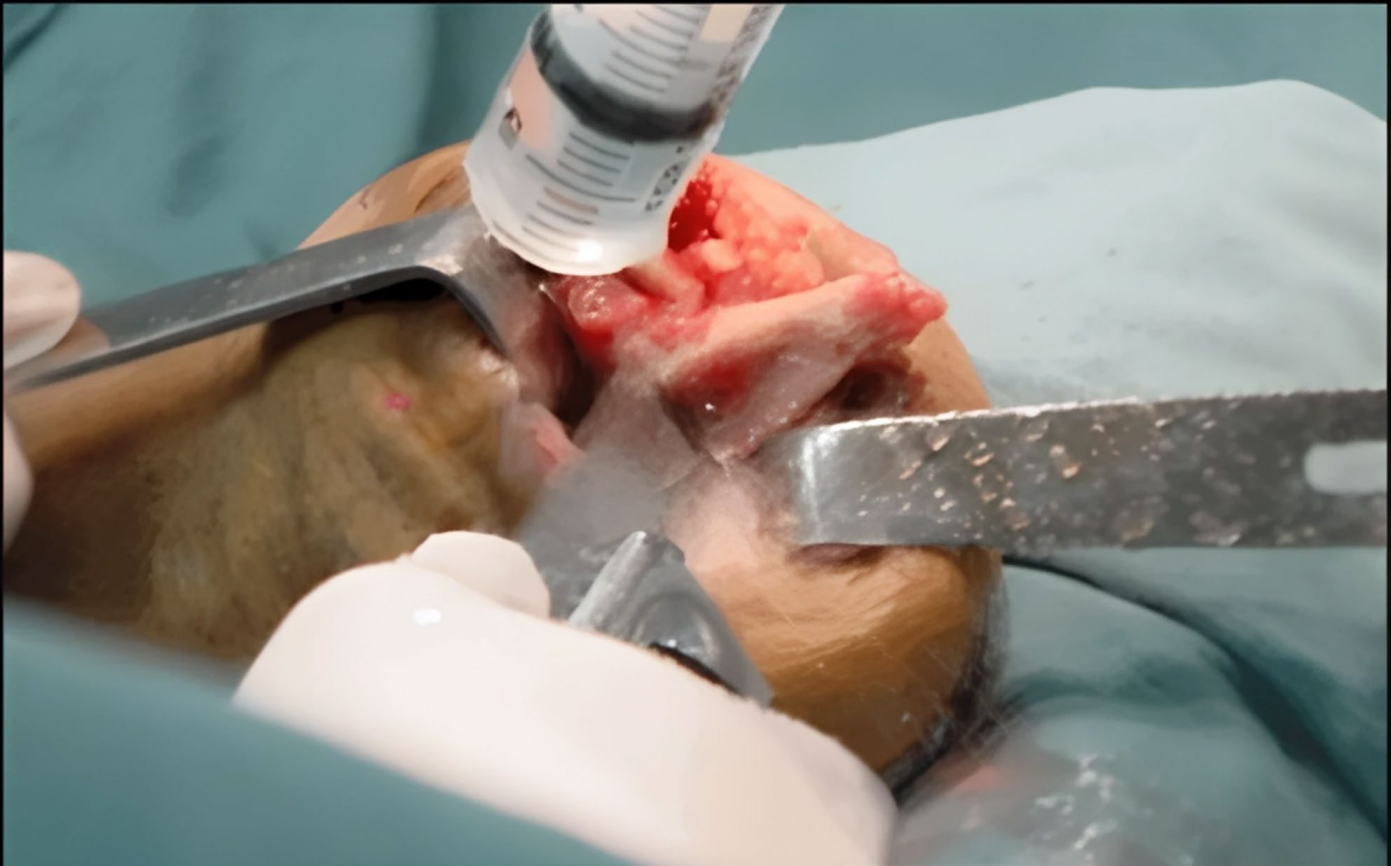
Image showing the bony prominence

Postoperatively, patients were managed and followed by a multidisciplinary team consisting of an orthopaedic surgeon, physiotherapist, and prosthetic technician. Prosthetic refitting and gait rehabilitation were initiated after wound healing, with regular follow-up visits to ensure symptom resolution, prosthetic tolerance, and functional ambulation. (Figs. 1 and 2).

### Statistical analysis

Statistical analyses were performed using IBM SPSS Statistics for Windows, Version 26.0 [19] Continuous variables are presented as means ± standard deviations, while categorical variables are presented as frequencies and percentages. Associations between categorical variables, such as residual limb length categories and stump complications, were analyzed using the chi-square test. A two-tailed p-value of less than 0.05 was considered statistically significant.

## Results

A total of 110 patients underwent surgical revision of 120 transtibial amputation stumps secondary to war-related trauma. The cohort comprised 77 males (70.0%) and 33 females (30.0%), with a mean age of 27.3 ± 11.2 years (range: 5–65 years). The mean interval between the initial injury and stump revision surgery was 25.8 months.

### Laterality of amputation

Among the 110 included patients, 100 had unilateral transtibial amputation and 10 had bilateral amputations, resulting in a total of 120 revised stumps.

### Stump length classification

Residual stump length distribution among the 120 stumps is summarized in Table 1.

**Table 1:** Stump length classification (*N* = 120 stumps)

Stump Length	Number of Cases	Percentage (%)
Very short	12	10.00
Short	35	29.17
Optimal	53	44.17
Long	15	12.50
Syme amputation	5	4.17

Note: Percentages were calculated based on the total number of stumps (*N* = 120)

### Etiology of amputation

Mechanisms of injury are summarized in Table 2.

**Table 2:** Etiology of amputation (*N* = 120 stumps)

Cause of Injury	Number of Cases	Percentage (%)
Shelling	85	70.83
Landmine explosion	20	16.67
Gunshot injury	15	12.50

Note: Percentages were calculated based on the total number of stumps (*N* = 120)

### Primary Stump-Related complications

The distribution of complications requiring revision surgery is summarized in Table 3. Some stumps presented with more than one complication.

**Table 3:** Primary Stump-Related complications (*N* = 120 stumps)

Complication	Number of Cases	Percentage (%)
Bone spur	55	45.83
Neuroma	27	22.50
Excessive soft tissue	13	10.83
Scar formation	13	10.83
Ulceration	12	10.00

Note: Percentages were calculated based on the total number of stumps (*N* = 120). Some stumps presented with more than one complication

### Relationship between stump length and complications

The distribution of stump complications by residual limb length is presented in Table 4. Percentages in parentheses are calculated based on the total number of stumps (*N* = 120). Symptomatic neuromas were significantly more frequent in short and very short stumps (χ² test, *p* = 0.010). In contrast, bony prominences were significantly more common in Syme and long stumps (χ² test, *p* = 0.030).

**Table 4:** Relationship between stump length and complications (*N* = 120 stumps)

Stump Length	Neuroma (*n* = 27)	Bone Spur (*n* = 55)	Ulcer (*n* = 12)	Scar (*n* = 13)	Excess Soft Tissue (*n* = 13)	Total Cases
Very short	7 (5.83)	2 (1.67)	0 (0.00)	1 (0.83)	2 (1.67)	12
Short	16 (13.33)	12 (10.00)	2 (1.67)	2 (1.67)	3 (2.50)	35
Optimal	4 (3.33)	23 (19.17)	10 (8.33)	8 (6.67)	8 (6.67)	53
Long	0 (0.00)	13 (10.83)	0 (0.00)	2 (1.67)	0 (0.00)	15
Syme	0 (0.00)	5 (4.17)	0 (0.00)	0 (0.00)	0 (0.00)	5

Note: Percentages in parentheses are calculated based on the total number of stumps (*N* = 120). Significant associations: Symptomatic neuromas were significantly more frequent in very short/short stumps (χ² test, *p* = 0.010). Bony prominences were significantly more common in Syme/long stumps (χ² test, *p* = 0.030)

### Relationship between cause of injury and complications

The relationship between mechanism of injury and stump complications is presented in Table 5. Percentages in parentheses are calculated based on the total number of stumps (*N* = 120). Ulceration and scar formation were significantly more common in landmine-related amputations (*p* = 0.010 for ulcers; *p* = 0.030 for scars). There was no significant association between mechanism of injury and neuroma formation (*p* = 0.321) or bony prominence (*p* = 0.311). Excess soft tissue showed a borderline non-significant association with mechanism of injury (*p* = 0.098).

**Table 5:** Relationship between cause of injury and complications (*N* = 120 stumps)

Cause of Injury	Neuroma (*n* = 27)	Bone Spur (*n* = 55)	Ulcer (*n* = 12)	Scar (*n* = 13)	Excess Soft Tissue (*n* = 13)	Total Cases
Gunshot injury	4 (3.33)	3 (2.50)	0 (0.00)	0 (0.00)	8 (6.67)	15
Landmine explosion	0 (0.00)	1 (0.83)	12 (10.00)	7 (5.83)	0 (0.00)	20
Shelling	23 (19.17)	51 (42.50)	0 (0.00)	4 (3.33)	5 (4.17)	85
p value	0.321	0.311	0.010	0.030	0.098	

Note: Percentages in parentheses are calculated based on the total number of stumps (*N* = 120)

*p* values are reported to three decimal places

Significant associations: Ulceration and scar formation were significantly more common in landmine-related amputations (*p* = 0.010 and *p* = 0.030, respectively). No significant association was observed for neuroma (*p* = 0.321) or bone spur (*p* = 0.311). Excess soft tissue showed borderline non-significant association (*p* = 0.098).

### Prosthetic rehabilitation time

The average time from stump revision surgery to successful prosthetic fitting ranged from 95 to 140 days. Successful prosthetic fitting was defined as pain-free tolerance of the prosthesis with functional ambulation during daily activities following gait rehabilitation. Rehabilitation was faster in younger patients and in those with optimal stump length.

## Discussion

### General considerations

This study presents outcomes of transtibial stump revision in a war-related cohort from Syria. The Syrian conflict created a large population of traumatic amputees, many of whom underwent emergency amputations under austere conditions. Once clinical services stabilized, it became essential to systematically evaluate stump complications, revision patterns, and rehabilitation outcomes. Publishing these findings after the acute phase of the conflict is particularly important to preserve long-term surgical lessons that can inform amputation planning, revision strategies, and prosthetic rehabilitation in similar humanitarian crises.

### Demographic characteristics

The majority of our patients were young males (mean age 27.3 years; 70% male), reflecting the demographic most exposed to war-related trauma. Comparable trends have been reported in other conflict settings, where young male civilians and combatants are disproportionately affected by high-energy injuries such as blasts and gunshots [1]. In contrast, non-war civilian cohorts typically include larger proportions of older vascular amputees with comorbidities, which may partly explain differences in complication profiles and revision indications [2].

### Neuroma formation

Symptomatic neuroma was a leading indication for revision in our cohort and occurred significantly more often in short and very short stumps. This finding is consistent with imaging-based studies demonstrating neuroma rates reaching 79%, with the superficial fibular nerve being the most frequently involved [3]. Moreover, systematic reviews have estimated symptomatic neuroma incidence in lower-limb amputees between 19% and 34%, increasing with longer follow-up durations [4, 5].

In our cohort, neuroma pain was not universal, despite clinical signs such as localized tenderness and paresthesia, which aligns with ultrasonographic reports describing both symptomatic and silent neuromas [3]. Other cohorts reported only 4–5% symptomatic neuromas within the first year after amputation, suggesting that neuroma-related symptoms may develop later or remain clinically silent in some individuals [6]. These findings underscore the importance of long-term follow-up and the need for early detection strategies in vulnerable war-related amputee populations.

### Bone Spurs and bony complications

Bone spurs were the most common stump complication overall (45.8%) and were significantly more frequent in long and Syme amputations. Similar patterns have been described in the literature, where longer residual tibial length and irregular bone contour have been associated with spur formation and distal end-bearing intolerance [7]. While some imaging studies have suggested that neuromas may adhere to bony spurs and exacerbate pain [8], neither our findings nor recent cross-sectional analyses demonstrated a statistically significant neuroma–spur correlation [9]. This highlights that bony spurs may act primarily as a mechanical problem affecting socket tolerance, rather than being a direct indicator of neuroma-related pain.

### Ulceration, Scarring, and mechanism of injury

A distinctive feature of this cohort was the strong association between landmine injuries and chronic ulceration as well as problematic scarring. High-energy blast trauma often produces unstable soft tissue coverage, irregular wound edges, and contaminated tissue planes, which predispose to delayed healing and recurrent breakdown [10]. A Turkish cohort of landmine amputees also documented higher neuroma excision rates, with pain appearing on average 9.6 months after prosthetic use [11]. Together, these findings reinforce that in war trauma, the mechanism of injury is not only a predictor of amputation level but also a determinant of stump tissue quality and long-term revision needs.

### Rehabilitation outcomes and the “Optimal length Paradox”

The mean time to successful prosthetic rehabilitation after revision in our cohort was 95–140 days, and outcomes were generally better in younger patients and those with optimal stump length. Prior studies have shown that residual limb length strongly influences prosthetic success, as adequate tibial length improves leverage, stability, socket comfort, and reduces energy expenditure during ambulation [12]. Younger age similarly correlates with faster recovery due to better tissue healing capacity and physical resilience [13].

Interestingly, while neuroma incidence was lowest in the optimal-length group, this group still accounted for the highest absolute number of bone spurs and ulcers. This apparent paradox is likely explained by the fact that optimal-length stumps represented the largest subgroup in our cohort, thereby contributing the greatest absolute complication burden. In addition, optimal-length stumps allow greater prosthetic loading and activity, which may increase exposure to repetitive mechanical forces at the stump–socket interface, potentially predisposing to pressure-related ulcers and reactive bony changes. This observation supports the concept that “optimal length” improves functional potential, but requires meticulous soft tissue balancing and socket management to prevent secondary mechanical complications.

### Preventive surgical strategies and the role of advanced techniques

Advanced nerve-interface procedures such as Regenerative Peripheral Nerve Interface (RPNI) and Targeted Muscle Reinnervation (TMR) were not available during the study period, reflecting resource limitations typical of humanitarian surgical environments. The conflict setting and resource limitations prevented implementation of advanced nerve-interface techniques; therefore, revision surgery relied on standardized conventional methods that remain highly relevant for low-resource humanitarian practice.

Recent evidence supports the effectiveness of RPNI and TMR in reducing neuroma formation and post-amputation pain. Retrospective analyses have demonstrated smaller neuroma size and lower pain scores following RPNI compared with traditional nerve handling [14, 15], and prophylactic RPNI has been shown to markedly reduce neuroma pain and increase prosthetic tolerance in selected cohorts [16]. Importantly, however, the outcomes in this cohort highlight that even in the absence of advanced technologies, structured revision strategies—particularly proximal nerve ligation with muscle burial, careful bone contouring, and robust soft tissue management—can restore prosthetic use and function. Therefore, in conflict zones or low-resource settings, adherence to basic nerve-handling principles remains clinically valuable and may offer a pragmatic approach when RPNI/TMR are not feasible.

## Conclusion

This Syrian cohort highlights the distinctive profile of transtibial stump complications following war-related trauma. The predominance of young male patients, the strong association of neuroma with short stumps, the increased risk of bone spurs in long and Syme amputations, and the higher rates of ulceration and scarring after landmine injuries illustrate the complex interplay between residual limb anatomy, tissue viability, and injury mechanism.

Publishing these results after the conflict is crucial to preserve surgical insights gained under extreme humanitarian conditions and to inform both humanitarian and civilian amputation practice. The findings emphasize the importance of achieving appropriate stump length, meticulous nerve handling, careful bony contouring, and durable soft tissue coverage during primary amputation and revision surgery to optimize long-term rehabilitation outcomes.

### Future directions

Future work should focus on improving early detection and prevention strategies for stump-related morbidity. Key priorities include:

1. Routine ultrasound surveillance for early identification of neuroma formation and stump pathology.
2. Integration of RPNI or TMR into revision protocols where feasible and sustainable within humanitarian systems.
3. Long-term multicenter follow-up studies evaluating prosthetic use, chronic pain trajectories, quality of life, and return-to-work outcomes in war-related amputees.

By applying these lessons, surgical teams working in conflict and post-conflict settings may significantly improve long-term function and quality of life among amputees.

## Data Availability

The data used in preparing the research was obtained with the permission and consent of the Syrian Arab Red Crescent Clinic and is not intended for public publication and is available upon request after obtaining permission to participate.
